# Using of aluminum (lignin /silica /fatty acids) hybrid filler in the fabrication of natural rubber conductive elastomers

**DOI:** 10.1038/s41598-025-09065-0

**Published:** 2025-07-14

**Authors:** Doaa S. Mahmoud, Khlood S. Abdel Zaher, Salwa H. El-Sabbagh, A. M. Yossef, Galal A.M. Nawwar

**Affiliations:** 1 Polymers and Pigments Department, National Research Centre, 33 El-Bohouth Street, Dokki, Giza, 12622 Egypt; 2 Green Chemistry Department, National Research Centre, 33 El-Bohouth Street, Dokki, Giza, 12622 Egypt; 3 Inorganic Chemistry Department, National Research Centre, 33 El-Bohouth Street, Dokki, Giza, 12622 Egypt

**Keywords:** Electrical conductivity properties, Lignin hybrid filler, Natural rubber, Mechanical properties, Chemistry, Nanoscience and technology, Physics

## Abstract

Green flexible conductive composites (FCCs) with high flexibility and foldability have potential uses in wearables, artificial intelligence (AI), and other fields. This research explores the valorization of aluminum hybrid fillers (lignin, silica, and fatty acids) extracted from rice straw black liquor to develop sustainable rubber composites. The natural rubber (NR) matrix was reinforced with different fillers: sodium bentonite, silica, and a synthesized Al(LSF) hybrid filler. The blending was performed using a two-roll mill with certain working conditions. The characteristics of the Al(LSF) hybrid filler were analyzed in detail. The properties of mechanical, swelling, electrical conductivity and morphology of the synthesized rubber composites were assessed. Characterization revealed that Al(LSF) hybrid filler accelerates the vulcanization process of NR composites. Notably, the properties of the resulting composites, such as tensile strength, crosslink density, and reinforcement direction, are dependent on the filler grain size. Al(LSF) nanoparticles (< 40 nm) provide superior reinforcement due to their increased interfacial interaction with the NR matrix. Because of its better interaction and dispersion, the Al(LSF) hybrid filler exhibited more uniform distribution, according to SEM images. In contrast to sodium bentonite and silica, the Al(LSF)/NR composites exhibit improved electrical conductivity (σ) and dielectric permittivity (ε’). The addition of Al(LSF) to NR composites led to a pronounced increase in electrical conductivity (σ), reaching nearly 900% higher than that of the unfilled NR. The findings of this experiment are expected to facilitate the creation of economical and sustainable rubber composites for widespread use in rubber industries.

## Introduction

Conductive composites (CCs) are widely used in electronic clothing, human-machine interactions, and flexible electrodes due to their superior adaptability and conductivity^1^. CCs are a material consisting of conductive fillers embedded in a nonconductive matrix. Among them, flexible conductive composites (FCCs) are composed of conductive fillers distributed in elastomer and have a far higher mechanical flexibility than hard metal materials. FCCs have many benefits, such as massive reversible deformation, quick reaction, affordability, and ease of processing. Therefore, it is necessary to look for flexible polymer materials with strong breakdown strength, a tiny loss tangent, and large electric conductivity. Because of their capacity to sustain electrical conductivity despite mechanical deformation, FCCs have demonstrated attractive uses in electronic applications^2^. Natural rubber (NR) is a popular biopolymer matrix for FCCs due to its exceptional flexibility and deformability^3^.

Most FCCs are typically formed by integrating a single type of conductive filler, such as graphene, carbon black, and carbon nanotubes, in a flexible polymer matrix.

Creating most flexible conductive composites (FCCs) involves blending one type of conductive filler (like graphene, carbon black, or carbon nanotubes) into a flexible polymer matrix. To achieve good electrical conductivity, a large quantity of the conductive material is typically needed to create a continuous pathway throughout the matrix. However, using so much filler often leads to negative outcomes, such as making the rubber weaker and less flexible, causing the conductive material to spread unevenly, and limiting the overall electrical conductivity that can be achieved^3^. Carbon black (CB) is common reinforcing filler used in the rubber industry, and it is widely known for its high electrical and thermal conductivity. Carbon black is now classified as a group 2B carcinogen by the International Agency for Research on Cancer (IARC)^4^.

Carbon black production and use cause major health and environmental issues, highlighting the need for sustainable alternatives in the rubber industry. Renewable plant-based fibers are promising reinforcing fillers due to their low cost, biodegradability, ease of use, and good mechanical properties. A significant agricultural byproduct, rice straw, is often burned after harvest, a practice with serious environmental and health consequences. This open burning releases harmful pollutants, contributing to air pollution, climate change, and respiratory problems^5,6^.

Furthermore, researchers are actively seeking sustainable solutions by studying how to extract and understand the valuable components within rice straw^7–10^ to identify potential industrial applications, such as in paper production^11^, the rubber industry^12–16^, paints^17,18^, and agriculture^19^. Our scientific program emphasizes the recycling of waste. Specifically, we focus on developing innovative complexes derived from rice straw pulping black liquor. These complexes possess valuable functional groups, making them ideal for use as multifunctional components in rubber applications. These complexes are composed of several novel resources, including lignin and silica, which are abundant, inexpensive, biodegradable, and lightweight. Our prior research has focused on integrating these complexes into rubber composites. For instance, lignin/silica and calcium lignate/calcium silicate derived from rice straw have been evaluated as antioxidants in styrene-butadiene rubber (SBR) composites. These bio-based additives not only demonstrated antioxidant activity but also led to improved physical properties compared to conventional antioxidants like TMQ or IPPD^12^.

Furthermore, Abdel Zaher et al., have successfully extracted a zinc complex from rice straw containing lignin, silica, and fatty acids. This complex has shown potential as both an activator and an antioxidant in rubber composites^13^. In another study, Othman et al., studied the utilization of a copper complex (Cu(LSF) complex) derived from rice straw which has electrical conductivity properties in acrylonitrile-butadiene rubber (NBR) and also acts as an antioxidant, hardening agent, and fluid resistance enhancer^14–16^. Hassarutai et al. investigated the use of fly ash (FA), a waste material, as an activator in tire tread rubber rather than using zinc oxide (ZnO) as an activator which reduces the harmful impact of using ZnO on the environment^20^.

This manuscript specifically focuses on enhancing the electrical conductivity of NR composites. The aluminium (lignin/silica/fatty acids) hybrid filler was prepared from rice straw pulping black liquor to fabricate Al(LSF)/NR composites. The surface morphology of the Al(LSF) hybrid filler and its distribution in the NR matrix were examined by scanning electron microscopy (SEM). Using TGA, XRD, and FTIR, the chemical constitution and structure of the Al(LSF) surface were identified. To fully understand how the Al(LSF) affects the rhometric, mechanical, and dielectric properties of the NR-based FCCs, we explored the NR composites in relation to the loading hybrid fillers. Our findings could introduce novel hybrid filler for designing elastomer composites with exceptional conductivity.

## Experimental section

### Materials

Natural rubber of the SMR-20 type, density (0.913 g/cm^3^), glass transition temperature Tg = -75ºC, and Mooney viscosity ML (1 + 4) at 100ºC = 60–90, was graciously supplied by the transport and engineering company (TRENCO), Alexandria. The commercial-grade product polymerized 2, 2, 4-trimethyl-1, 2-dihydroquinoline (TMQ) as an antioxidant, stearic acid and zinc oxide (ZnO) as activators; naphthenic processing oil as a plasticizer; N-cyclohexyl-2-benzothiazole sulphenamide (CBS) as an accelerator; and elemental sulfur as a curing agent. These were provided by Aldrich Company, Germany. Sodium bentonite was provided by Alfa Aesar and Co. (Kandel, Germany). White powder (Hi-Sil 233D) was provided by PPG Industries Inc. (Delfzijl, Netherlands). Every commercial-grade rubber additive was used precisely as supplied.

### Preparation of Al(LSF) hybrid filler

**Scheme 1:**
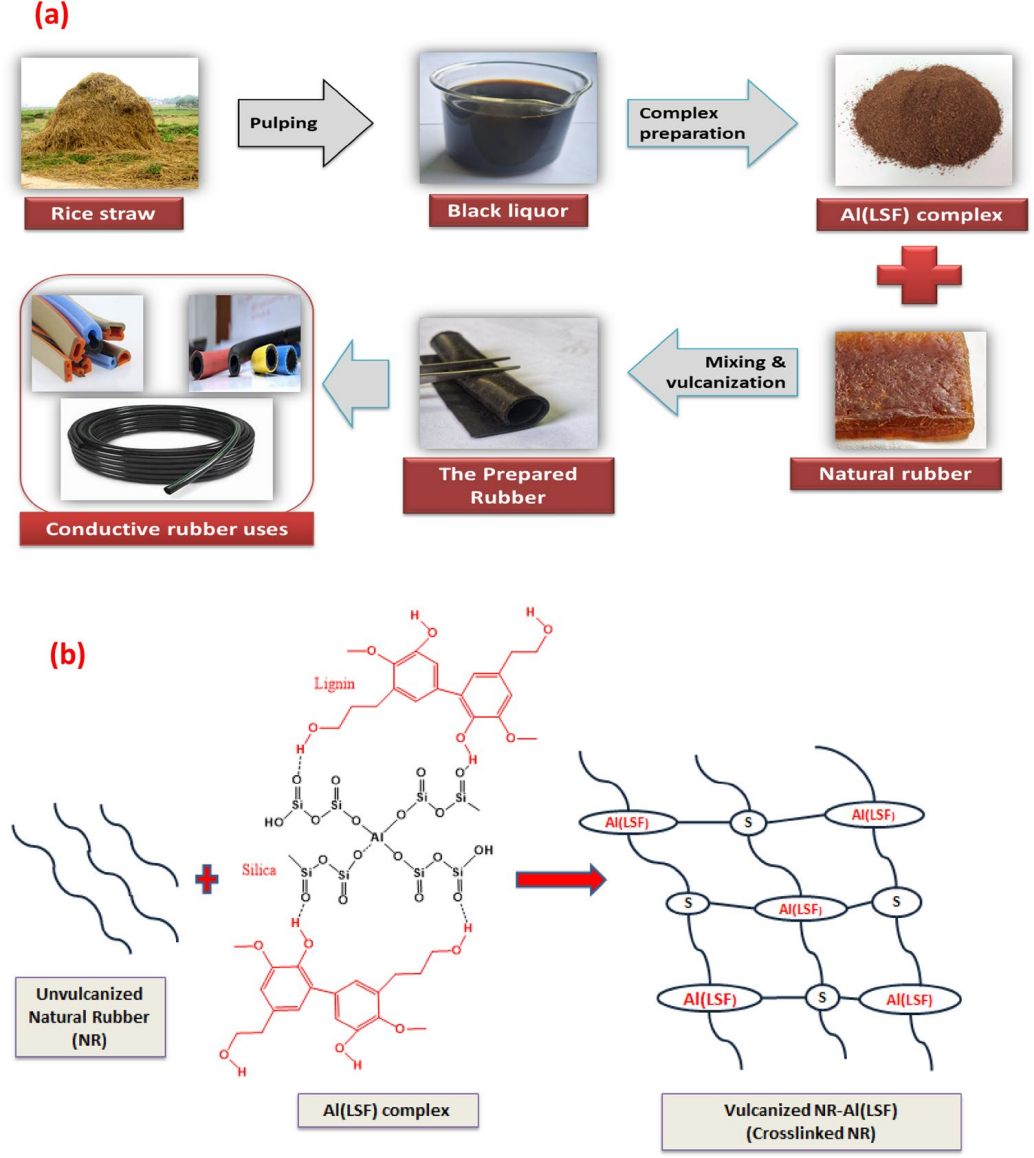
A schematic diagram (**a**) the preparation of conductive rubber using Al-LSF complex, (**b**) formation of crosslinking Rubber schematic representation of the proposed reaction mechanism for Al(LSF)-NR vulcanization, showing the interaction pathways involved.

Thirty grams of Al_**2**_(SO_4_)_**3**_.16 H_**2**_O were added to one liter of rice straw pulping black liquor (pH 12) while stirring. After the resulting liquid (pH 4) was allowed to stand overnight, 37 g of dark brown powder were obtained by filtering the precipitate, washing it with tap water, and oven-drying it at 105 °C (as shown in Scheme 1). As we previously described, the effluent was used^10,17^.

### Compounding and vulcanization of rubber composites

To create raw natural rubber with different concentrations of Al(LSF), bentonite, or silica, a straightforward and efficient two-roll mill mixing technique was employed. NR was first masticated for five minutes. Accordingly, NR (ASTM D 3184-89) was thoroughly mixed with various ratios of the studied fillers (10, 15, and 20 phr (parts per hundred rubbers)), paying particular attention to the uniform dispersion of all fillers in the NR matrix (Table 1). The earlier study^21^ reported the rule of mixture method.

**Table 1:** Formulation of NR composites.

Ingredient (phr)	NR_0_	NR-A_1_	NR-A_2_	NR-A_3_	NR-B_1_	NR-B_2_	NR-B_3_	NR-S_1_	NR-S_2_	NR-S_3_
AL-LSF complex	--	10	15	20						
Sodium bentonite	--				10	15	20	--	--	--
Commercial Silica	--	--	--	--	--	--	--	10	15	20
Rheometric characteristic at 152 ± 1 °C										
M_L_, dNm	0.46	0.34	0.35	0.48	0.91	1.07	1.47	0.61	0.52	0.6
MH, dN.m	10.65	8.34	8.55	11.26	12.37	15.53	10.04	10.81	9.09	9.92
ΔM, dN.m	10.19	8.02	8.2	10.78	11.46	12.46	9.17	10.2	8.57	9.32
t_S2_, min.	3.33	5.73	5.84	3.33	3.86	3.94	5.36	3.34	3.72	3.35
t_C90_, min	12.05	11.21	11.60	12.12	9.73	10.61	21.27	8.78	12.07	11.33
CRI, min.-1	11.47	18.28	17.36	11.33	17.04	14.99	6.29	10.38	11.97	12.53

Base recipe (in phr): NR (natural rubber) 100; stearic acid 2; zinc oxide 5; CBS (N-cyclohexyl-2-benzothiazole sulfenamide) 1; processing oil 3; sulfur 3; M_L_, minimum torque; M_H_, maximum torque; T_s2_, scorch time at 2 torque units after minimum; T_C90_, optimum cure time (at 90% cure); CRI, cure rate index.

### Characterization

The chemical characteristics and functional groups of the examined filler and NR composites were characterized qualitatively. The FTIR spectra were obtained using a JASCO FTIR-6000 E, (Japan). The examined filler was mixed with KBr (potassium bromide) to make discs, measured in the wave number range of 4,000–400 cm^− 1^, using a resolution of 4 cm^− 1^, and using Model ATR PRO450-S, Single Reflection Measuring Attachment for measuring NR composites.

A JEOL JEM 2100 transmission electron microscope (TEM) (Japan) equipped with a microanalyzer electron probe was used to study the material under examination. The surface of rubber composites was examined using a scanning electron microscope (SEM) and energy-dispersive X-ray analysis (EDAX) utilizing a Quanta FEG 250 connected with an EDAX unit. EDAX was used to identify elements on any compound’s surface. Philip’s diffractometer (Model PW1390) used Ni-filtered Cu Kα radiation (λ = 1.5404 Å) to obtain XRD at room temperature. A scan rate of 2°/min was used to measure the diffraction angle, or 2θ.

The Perkin Elmer analyzer equipment, USA, was used to perform the thermal gravimetric analysis (TGA) test. Sample weights ranging from 13 to 25 mg were scanned between 50 and 1000ºC at a heating rate of 10ºC/min and nitrogen airflow of 50 ml/min. NR mixtures were evaluated for rheometric characteristics using a Monsanto oscillating disc rheometer-100 (ASTM D 2084-07, 2007a). In a hydraulic press, compounded rubber was vulcanized at 4 MPa of pressure and 142 ± 1ºC for natural rubber.

When the samples are stretched to a certain extent and then allowed to retract at the same rate to the un-stretched state, the areas W_1_ (work completed during extension) and W2 (work done through regression) are used to calculate the hysteresis loss (Hls), which is the amount of energy dispersed through cyclic distortion, as expressed in Eq. (1):

1 $$\:HIs=\raisebox{1ex}{${W}_{1}$}\!\left/\:\!\raisebox{-1ex}{${W}_{2}$}\right.$$.

The swelling performance of NR loaded with different amounts of investigated filler was carried out in toluene according to ASTM D471-16a (2021).

The dielectric characteristics and electrical conductivity of the prepared compositions were measured in pellet form using an LCR meter (Hitester, model Hioki 3532-50, manufactured in Japan).

## Results and discussion

### Hybrid filler characterization

**Fig. 1:**
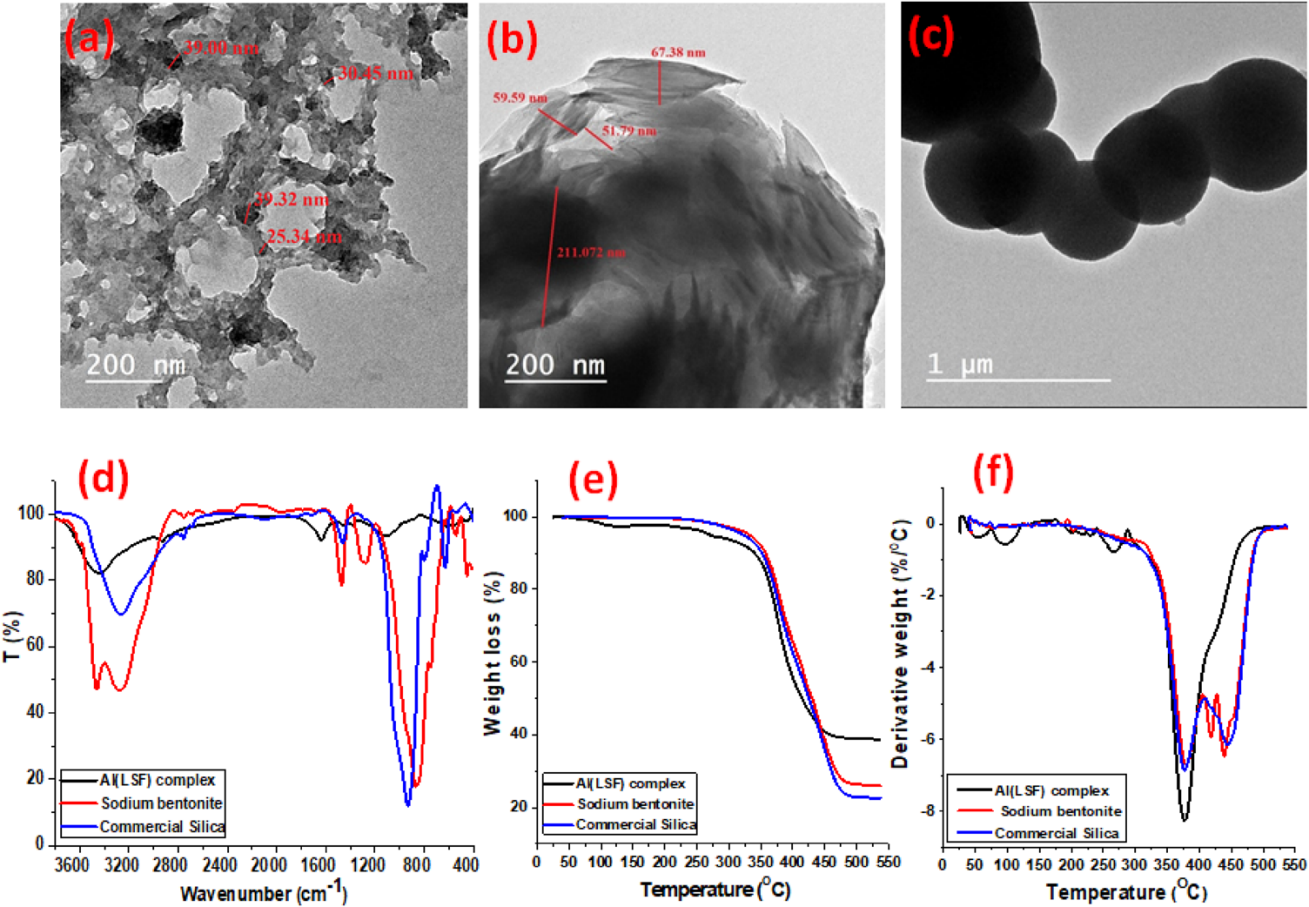
(**a**–**c**) TEM micrographs, (**d**) FT-IR, (**e**) TGA and (**f**) DTA for degradation of the investigated fillers.

The size, distribution, and structure of all the fillers under investigation were ascertained using the TEM technique. It is well known that the size of the particles has a significant impact on how they disperse inside the rubber matrix and, in turn, how well the rubber works mechanically. The AL(LSF) hybrid filler’s particle size is between 25 and 40 nm, as seen in Fig. 1(a). The lignin particles have a dark platelet shape, while the silica particles are brilliant spheres, with the two shapes overlapping^13^. The layered structure of sodium bentonite is depicted in Fig. 1(b), and its nanoscale particle size spans from 51 to 211 nm. The silica particles were spherical, as shown in Fig. 1(c), and the average size of commercial silica particles was between 52 and 73 μm. As though they were fibers tying the matrix together, these little particles reinforce it, creating a more uniform matrix.

The absorption bands of the Al(LSF) in the FT-IR spectra are shown in Fig. 1(d). A broad band appeared at 3444 cm^− 1^, which indicated the presence of stretching vibrations of the alcoholic and phenolic OH groups of lignin. Bands of 2931 cm^− 1^ appeared due to the presence of alkyl groups in the Al(LSF). The broad band at 1643 cm^− 1^ was attributed to the CO groups of fatty acids. The spectra of sodium bentonite showed the characteristic band at 3629 cm^− 1^, indicating the presence of OH stretching bands. Also, the intense band at 1037 cm^− 1^ indicates the presence of stretching vibrations in SiO_**2**_. In addition, broad bands of 3436 cm^− 1^ and 1643 cm^− 1^ are attributed to the –OH stretching mode of the interlayer water. The band at 1446 cm^− 1^ indicates the presence of stretching N–C. The spectra of silica showed the characteristic bands at 3436 and 1631 cm^− 1^ assigned to OH, as well as the bands at 1103 and 968 cm^− 1^ assigned to Si-O and Si-O-Si stretching vibration modes of Si-OH groups. The peak that appears at 802 cm^− 1^ may be caused by the O-Si-O vibration mode of SiO_2_^22^.

TGA and differential thermal analysis (DTA) were used to compare the thermal stability of the investigated fillers. Investigation of the fillers’ thermal degradation has been done in terms of percentage weight loss at various temperatures, as indicated in Fig. 1(e) and Fig. 1(f). The TGA curves of silica and Na-bentonite show similar single-stage degradation behavior, and the fillers’ first weight loss is approximately 80–100 °C as a result of moisture and other volatile substances evaporating. While the Al(LSF) hybrid filler showed the three decomposition stages, the first stage happened below 100 °C due to the evaporation of adsorbed water and other volatile matter. The second decomposition stage occurred between 100 and 285 °C, attributed to the chemical cleavage of fatty acids and the degradation of the propanoid side chain of the lignin in the complex. The greater third mass loss, which occurred between 320 and 446 °C, is associated with the thermal degradation of lignin inside the complex, namely the β–β and C–C linkages that connect the lignin basic units. The residual weight of the Al(LSF) is about 38.6 wt%, but the residual weight of Na-bentonite and silica is about 25.9 wt% and 22.5 wt%, respectively. These findings demonstrated the superior thermal stability of the Al(LSF) over silica and Na-bentonite due to the cross-linking of lignin and fatty acids with silica in the Al(LSF)^26^.

### Scanning electron microscope (SEM) and X-ray diffraction of the Al(LSF) hybrid filler

**Fig. 2:**
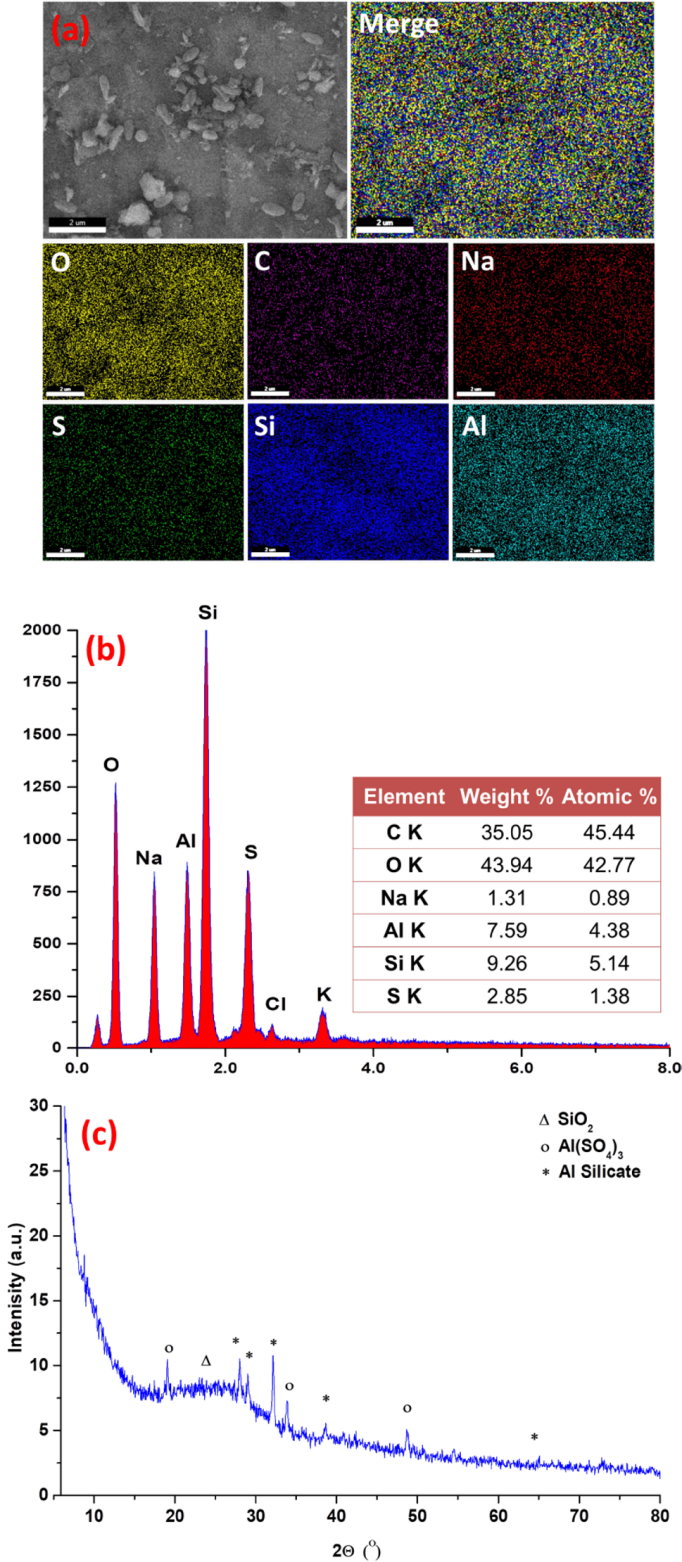
SEM, EDX, mapping and XRD pattern of Al(LSF) complex.

A scanning electron microscope (SEM) generates high-resolution images by scanning a sample’s surface with a focused electron beam. The interactions between the beam and the sample provide information about the surface topography and composition. Scanning electron microscope (SEM) of Al(LSF) hybrid filler indicates that clusters of silica and aluminium were properly distributed throughout the base lignin matrix Fig. 2(a). The EDAX analysis showed that the organic content of the Al(LSF) complex was C 24.74% wt. (representing the lignin and fatty acids), O 46.95% wt., Al 8.83% wt., Si 10.95% wt., S 3.38% wt., and Na 5.17% wt. Also, the EDAX mapping of the Al(LSF) complex shows a uniform distribution of Al, Si, C, O, Na, and S atoms, which indicates crosslinking between them Fig. 2(b)^23^.

In Fig. 2(c), X-ray diffraction of the Al(LSF) hybrid filler with the 2θ diffraction angle in the range of 10°-80° revealed many crystalline peaks with a large hump between 2θ = 15° and 30°, which may be assigned to the amorphous silica nanoparticles from the neat black liquor. This characteristic amorphous silica nanoparticle peak overlapped with new crystalline peaks at 2θ values of 19.071°, 28.038°, 29.018°, 32.132°, 33.902°, 38.616°, 48.692°, and 72.914°. A plausible explanation for the observed crystalline nature, as revealed by XRD, is the formation of a novel crystalline matrix. This matrix likely results from crosslinking interactions among the primary constituents: aluminum sulfate, silica, and lignin. Prior FTIR analysis and XRD provide supporting evidence for such crosslinking within the hybrid filler^24^.

#### Characterization of flexible conductive composites

##### FT-IR spectra

**Fig. 3:**
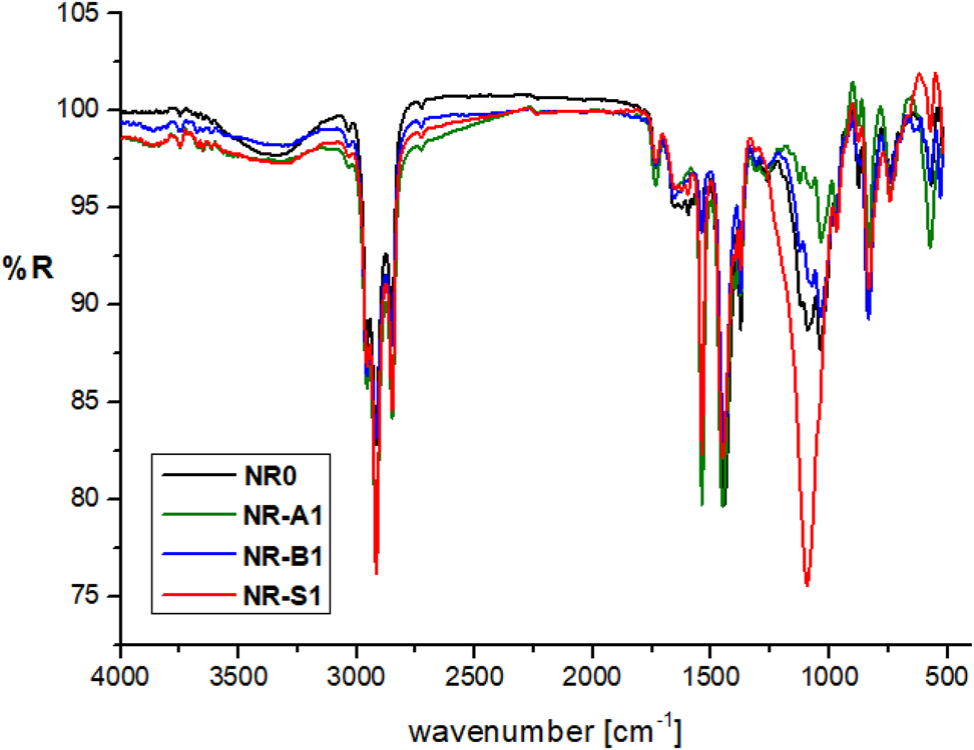
FT-IR of NR composites.

The broad band at 3436 cm^− 1^ attributed to water hydroxyl groups, which indicate the hydrophilicity of the Al(LSF) hybrid filler, appeared with a low-intensity peak in the IR spectrum of NR, with the complex at 3366 cm^− 1^ due to the hydrophobicity of NR, which indicates the absence of a characteristic hydroxyl signal in its spectrum (see Fig. 3). The strength of the peak at 2921 cm^− 1^ in the composite spectrum increases when both aliphatic bands are present in the parent NR and Al(LSF). The characteristic bands’ intensity altered when the Al(LSF) complex was introduced to NR, suggesting that the Al(LSF) was chemically bound to the rubber matrix. Alike observations have been noted in the case of NR/Zinc (Lignin/Silica/Fatty Acids)^13^.

#### SEM

**Fig. 4:**
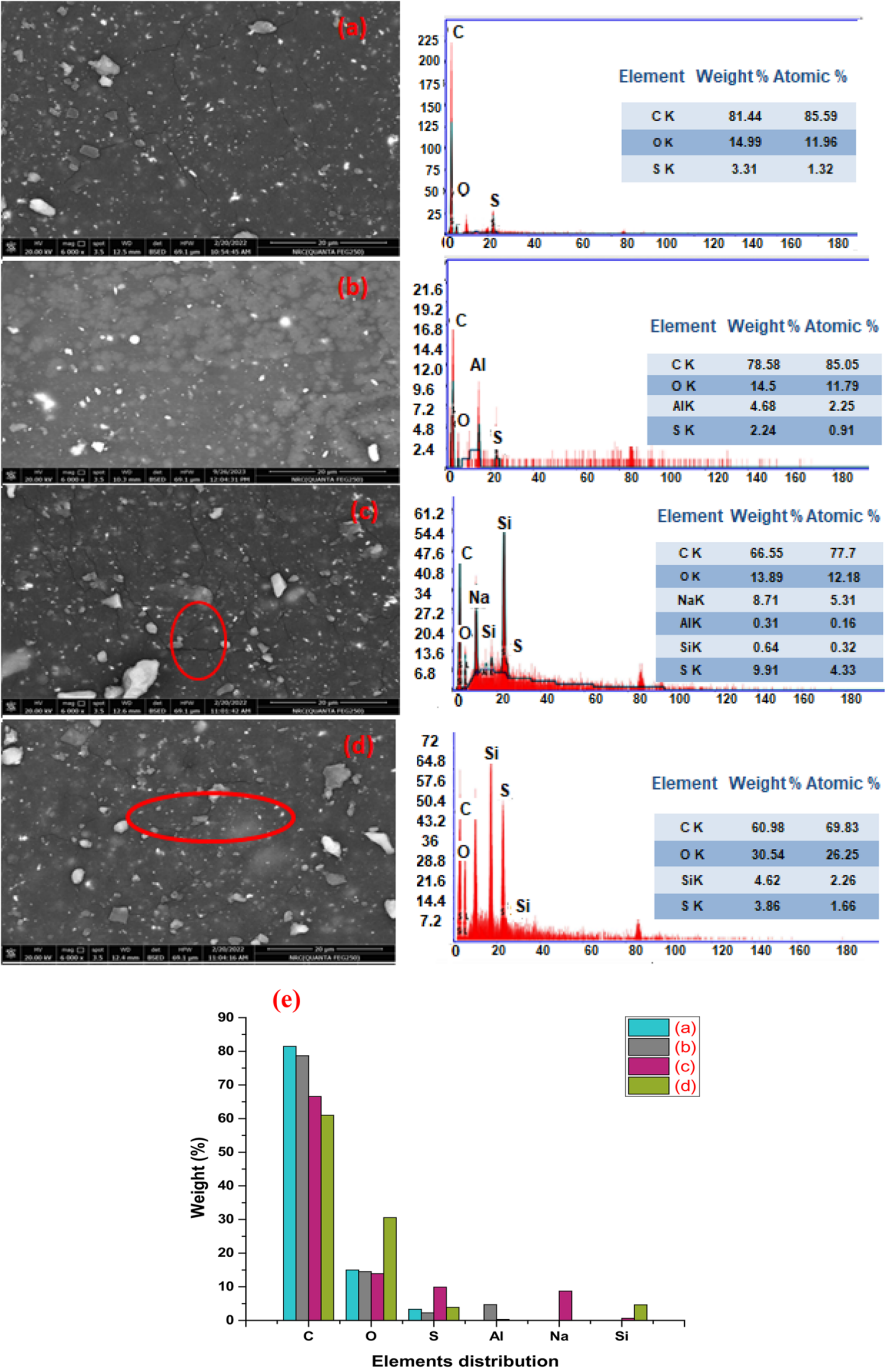
SEM-EDX of (**a**) NR-0, (**b**) NR-A_1_, (**c**) NR-B_1_, (**d**) NR-S_**1**_ and (**e**) EDX-based weight% distribution of compositional elements.

The distribution of fillers that affect the properties of natural rubber was identified using SEM. The surface of the control sample (NR-0), as shown in Fig. 4(a), exhibited typical surface morphologies with perturbations, as would be expected from rubber composites filled with carbon black. However, white particles in the NR-0 are identified as ZnO and sulfur by EDAX in Fig. 4 (a). The Al(LSF) hybrid filler is found to be finely distributed in the NR matrix, and the absence of particle detachment suggests that the filler is effective at promoting rubber adhesion. Figure 4 (c) and (d) display a microphotograph of the NR-B_1_ and NR-S_1_ composites, where we can see an irregular surface with cracks of the sodium bentonite and commercial silica particles from the elastomeric matrix. This fact could point out that bentonite and silica have poor adhesion with the polymer matrix. Surface cracks in NR-B_1_ and NR-S_1_ composites are caused by weak interaction between the rubber compound phases and the filler particles.

This is caused by the hydrophilic hydroxyl groups found in silica and bentonite, which have a tendency to form hydrogen bonds with other polar chemical materials in the rubber matrix. The observations supported the decrease in tensile strength of NR-B_1_ and NR-S_1_ composites. Figure 4(b) shows NR-A_1_ has stronger filler-rubber interactions than other vulcanizates at low loading. Because the nano-sized Al(LSF) may create a large contact surface for interaction with rubber chains and can be evenly dispersed throughout the rubber matrix. Furthermore, lignin’s aromatic rings may interact with rubber molecules’ alkenes to produce further reinforcement^25^. Also, the presence of fatty acids in the Al(LSF) provides better complex dispersion, which leads to significant improvements in the mechanical and dielectric properties. The overall element composition of each sample was estimated using EDAX analysis. Depending on the samples, different mineral atoms were observed. The NR-0 sample contained several elements, including C with a peak area of 81.44% wt., O with a peak area of 14.99% wt., and S with a peak area of 3.31% wt. The NR-A_1_ sample showed the presence of C 78.58% wt., O 14.5% wt., Al 4.68% wt., and sulfur 2.24% wt. The NR-B_1_ sample was dominated by several elements, including C (66.55% wt), O (13.89% wt), Na (8.71% wt), Al (0.31% wt), Si (0.64% wt), and S (9.91% wt). The NR-S_1_ sample showed the presence of C (60.98% wt), O (30.54% wt), Si (30.54% wt), and S (3.86% wt)^26^.

#### TGA

**Fig. 5:**
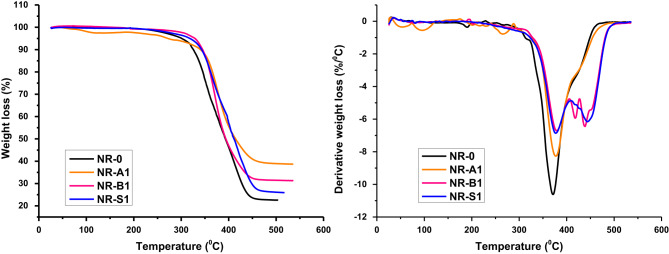
TGA and DTA for degradation of NR-0; NR-A_1_; NR-B_1_ and NR-S_1_ composites.

The TGA and differential thermal analysis (DTA) values for the NR vulcanizates are shown in Fig. 5. Every sample shows the same single-stage degradation pattern, with moisture and other volatile elements evaporating to cause an initial weight loss of about 80–100 °C. At the starting temperature of 260 °C, the samples primarily begin to deteriorate. The first breakdown of compounding elements such as stearic acid, zinc oxide, accelerators, and antioxidants was caused by continuous chain scission and cross-linking. As indicated in Table 2, the results demonstrated that the addition of silica, bentonite, and Al(LSF) hybrid filler enhanced the degradation resistance of NR composites.

**Table 2:** Thermo-gravimetric behavior of NR composites.

Sample codes	T_onst_°C	T _50_^o^C	T_max_^o^C	Peak temp. T_max_ (°C)	Residual weight (%)
NR-0	254	391.76	497.74	376.95	22.5
NR-A_1_	305	414.68	523.99	377.05	38.66
NR-B_1_	280	393.99	528.09	379.27	30.6
NR-S_1_	258	409.89	512.13	379.59	25.9

As seen in Fig. 5, a thermal stability index for rubber composites is thought to be the degradation temperature at 50% weight loss (T_50_)^27^. The TGA peak (T_50_) shifted to higher temperatures with the addition of the Al(LSF), from 391.67 °C to 414.68 °C. This shift was greater than that observed with bentonite (393.99 °C) and silica (409.89 °C), suggesting that the Al(LSF)-filled vulcanized rubber had increased thermal stability. Because the Al-complex filler has good thermal stability and heat resistance, it delays the thermal deterioration of natural rubber, which explains why the decomposition temperature for the Al(LSF) was higher at 50% wt% than for bentonite and silica. The NBR/Cu-LSF combination demonstrated thermal stability in this finding, which is consistent with earlier research, and the Cu-LSF complex served as a thermal stabilizer in this instance^14,15^.

A strong heat resistance is indicated by the increased T_50_, which suggests that the Al(LSF) complex has improved the resistance to thermal degradation. This is explained by the high thermal stability of silica and the complex’s anti-oxidative properties, via the phenolic hydroxyl groups in lignin, which inhibit oxidation. The active centers of the NR rubber leading chains’ degradation were most likely inactive when they encountered the filler, and interactions between the Al(LSF) complex and rubber increased the chemical and physical cross-linking points, delaying further breakdown of rubber chains^28^. As demonstrated by Table 2, NR composites filled with silica and bentonite exhibit smaller decomposition residues than NR composites filled with the Al(LSF). Additionally, NR composites have stronger filler-matrix interaction, as indicated by the greater residue for the Al(LSF). Furthermore, the larger residue for the Al(LSF) in NR composites suggests a stronger filler-matrix connection. Rubber compounds having better filler-matrix interactions generated more breakdown residues, according to Ismail et al.^29^.

#### Curing characteristics

Curing properties play an essential role in understanding the process ability of rubber compounds because they determine the degree of cross-linking in relation to temperature and curing time. The time before vulcanization is termed “scorch time” or “induction time.” The degree of cross-linking or curing time needed to prepare the vulcanized NR is represented by the optimum cure time. Processability is correlated with minimum torque, whereas rubber compound stiffness and cross-link density are correlated with maximum torque. The curing behaviors of the NR composites with various Al(LSF) hybrid filler, Na-bentonite, and silica contents are summarized in Table 1.

For the Al(LSF)/NR composites, the torque values decrease with an increasing Al-complex content and then increase. Such a decrease indicates worse interfacial adhesion between lignin (present in the Al(LSF) and rubber matrix segments^29^. Nonetheless, the researchers found that lignin was evenly distributed throughout natural rubber, increasing the compound’s resistance to scorch and enhancing the optimum cure time. Na-bentonite and/or commercial silica/NR composites showed a slight increase in torque values when compared to Al(LSF)/NR composites^30^. The NR composites show a greater value of M_H_ and ∆M (torque difference) with increasing Al(LSF) content, indicating greater physical crosslinking between the Al-complex and the NR matrix. This might be caused by the formation of a filler-rubber link among the complex and NR matrix, as well as the increased cross-link density in Al(LSF)-filled NR compounds.

The findings indicate that an increase in filler loading causes a decreasing trend in the scorch time (t_s2_) and cure time (t_c90_) of Al(LSF), Na-bentonite, and silica-filled NR composites, respectively. The vulcanization of rubber was postponed by the addition of Al-hybrid filler to the rubber matrix. This was because the complex phenol groups in lignin inhibited the effects of radical scavenging. Shorter scorch times typically mean that the cross-linking reaction began earlier. The lower-filled loading in NR compounds shows lesser or shorter t_s2_ and t_c90_ compared to unfilled NR compounds^30,31^.

#### Mechanical properties

**Fig. 6:**
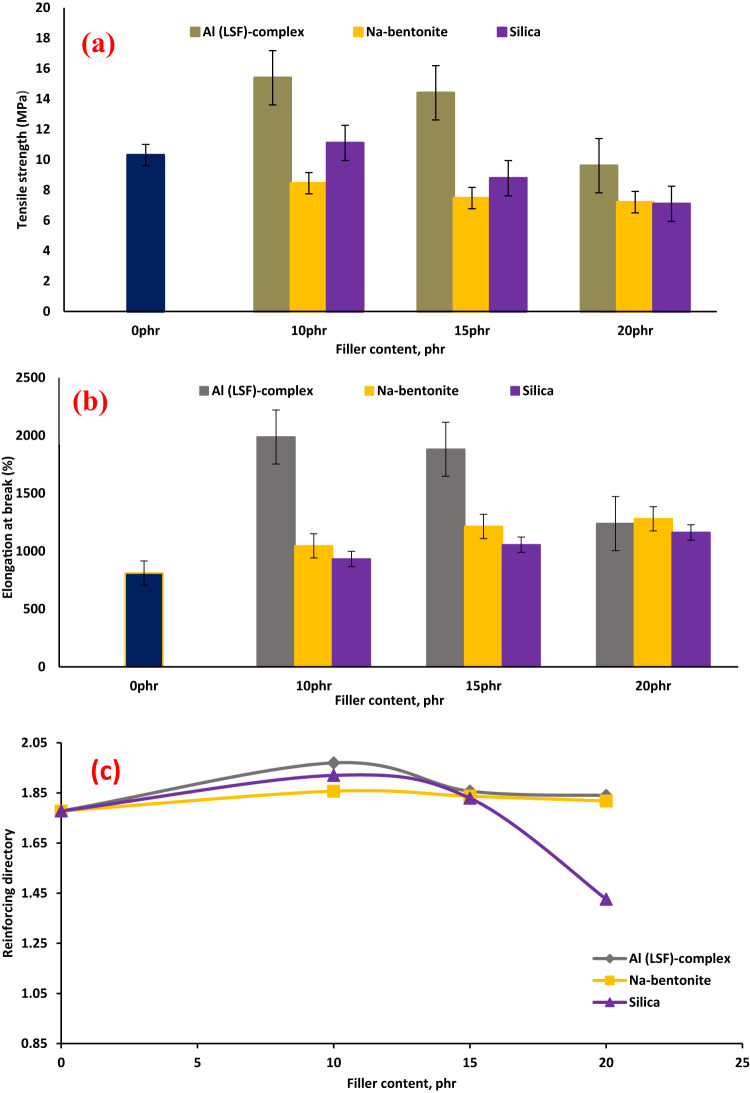
Tensile properties: (**a**) tensile strength; (**b**) elongation at break and (**c**) reinforcing directory of NR composites.

Figure 6 summarizes the mechanical characteristics of the NR composites as a function of filler loading. Compared to pure NR (i.e., the control sample) and other produced composites, the tensile strength of the Al(LSF)/NR composites increased even more. SEM micrographs reveal that the Al(LSF) hybrid filler has a reinforcing impact on tensile strength due to its greater specific surface area and smaller particle size when compared to silica and Na-bentonite fillers^32,33^. The strength of Al(LSF), Na-bentonite, and silica filled NR increased at 10phr before eroding. Filler-filled NR composites showed initial strength improvements due to improved filler-matrix interactions, assisted by the platy fillers. External loading increases filler-matrix interactions, transferring more stress from the matrix to the fillers^34^.

Adding high content (20 phr) of Al(LSF), Na-bentonite, and silica to NR composites leads to reduced tensile strength due to filler agglomeration, resulting in insufficient particle homogeneity and hardness^33^. Figure 6(a) clearly shows that the elongation at break was relatively enhanced through the increase of Al(LSF), Na-bentonite, and silica loading content in all composites. The maximum elongation at break values of the NR composites were obtained by the Al(LSF) loading at 10 phr (as shown in Fig. 6(b). This could be attributed to the molecular chains of NR having a large number of double bonds, which combine with sulfur to form a network that strengthens and increases the elasticity of the rubber. On the other hand, the elongation at break begins to reduce at 20 phr (Al(LSF) also due to its aggregation in the NR matrix, which makes the composite more brittle^33^. Al(LSF)/NR composites displayed lower modulus at 100% and 300% elongation when compared to unfilled and other filled-NR composites^35^. This study shows promise for fillers as it does not decrease their flexibility while reinforcing rubber composites, like standard fillers such as carbon black and silica.

The “reinforcing index” or “reinforcing directory” quantifies filler’s effectiveness in enhancing the mechanical performance of polymeric materials, such as rubber and plastics. It is defined as the ratio of the modulus at 300% elongation (M₃₀₀) to the modulus at 100% elongation (M₁₀₀), as expressed in Eq. (2):

2 $$\text{Re} \inf orcing~index~\left( {RD} \right) = {\raise0.7ex\hbox{${M_{{300}} }$} \!\mathord{\left/ {\vphantom {{M_{{300}} } {M_{{100}} }}}\right.\kern-\nulldelimiterspace} \!\lower0.7ex\hbox{${M_{{100}} }$}}$$

This parameter provides insight into the reinforcing efficiency of the filler within the elastomeric matrix^29,36^. Furthermore, the reinforcing index presented in Fig. 6(c) exhibits an increasing trend for Al(LSF), Na-bentonite, and silica fillers up to a loading of 10 phr, followed by a decline with further increases in filler content^37–39^. This behavior can be attributed to the lack of surface modification agents, which may result in dominant filler–filler interactions rather than effective filler–rubber matrix interactions. Reinforcement is typically enhanced through strong interfacial bonding either physical or chemical between the filler and the rubber matrix, which facilitates efficient stress transfer and mechanical property improvement^40,41^. It is evident from Fig. 6 that the Al(LSF) performs better as filler for natural rubber than silica and Na-bentonite. One drawback of combining Na-bentonite and silica without a coupling agent is that adhesion and contact between the nonpolar rubber and the polar filler are weak^42^.

To understand the filler-rubber interactions inside the filled rubber composites, Bhowmick et al.^43–45^ employed a model Equation (3): 

3 $$\:{E}_{f}=\:\:A{E}_{0}{e}^{B\varphi\:}$$.

A novel generalized exponential formula was put forth. Connecting the filler’s modulus and volume fraction in the manner shown below, where A and B are thought to be constants for a particular strain level and temperature, and where f and 0 denote full and neat rubber samples. Where E is Young’s modulus (Young’s modulus measures the resistance of a material to elastic (recoverable) deformation under load), it is related to the filler volume percentage in the NR composites. Equation (4) has the following form in log scale:

4 $$\:ln\frac{{E}_{f}}{{E}_{0}}=lnA+B\varphi\:$$.

**Fig. 7:**
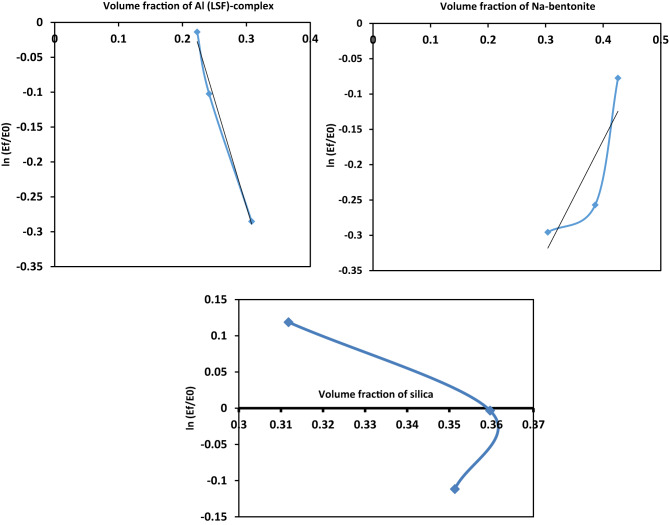
Variation of Young’s modulus versus volume fraction of NR composites.

Plotting ($\:ln\frac{{E}_{f}}{{E}_{0}}$) values versus various volume fractions of the filled composites under investigation is shown in Fig. 7. Then the slope of the straight line in this picture represents the constant values. The best-fit straight-line equation for the experimental values is represented in Eqs. (5, 6, 7) as follows:

For Al(LSF)

5 $$ln\frac{{{E_f}}}{{{E_0}}}=\ln \left( {0.65707} \right)+\left( {3.069344} \right)\phi ~~$$.

For Na-bentonite

6 $$ln\frac{{{E_f}}}{{{E_0}}}=\ln \left( {0.8116} \right)+\left( { - 1.5896} \right)\phi ~$$.

For silica

7 $$ln\frac{{{E_f}}}{{{E_0}}}=\ln \left( {0.66121} \right)+\left( {1.71075} \right)\phi ~$$.

The constant B describes the interactions between the filler and NR rubber in the composites^46^. It is observed that the Al(LSF) gives a bigger slope than Na-bentonite or silica. Through the formation of hydrogen bonds among the aluminum silicate layers and the NR matrix, the dispersion of aluminum silicate layers increases the modulus, resulting in intense contact. According to Helaly et al.^47^, there is a better interaction between rubber chains and the aluminum silicate surface that leads to the promotion of Young’s modulus, which in turn retards the mobility of NR rubber chains adjacent to the Al(LSF) surface. These results closely match the latter characteristics (mechanical and rheological).

#### Swelling studies

**Fig. 8:**
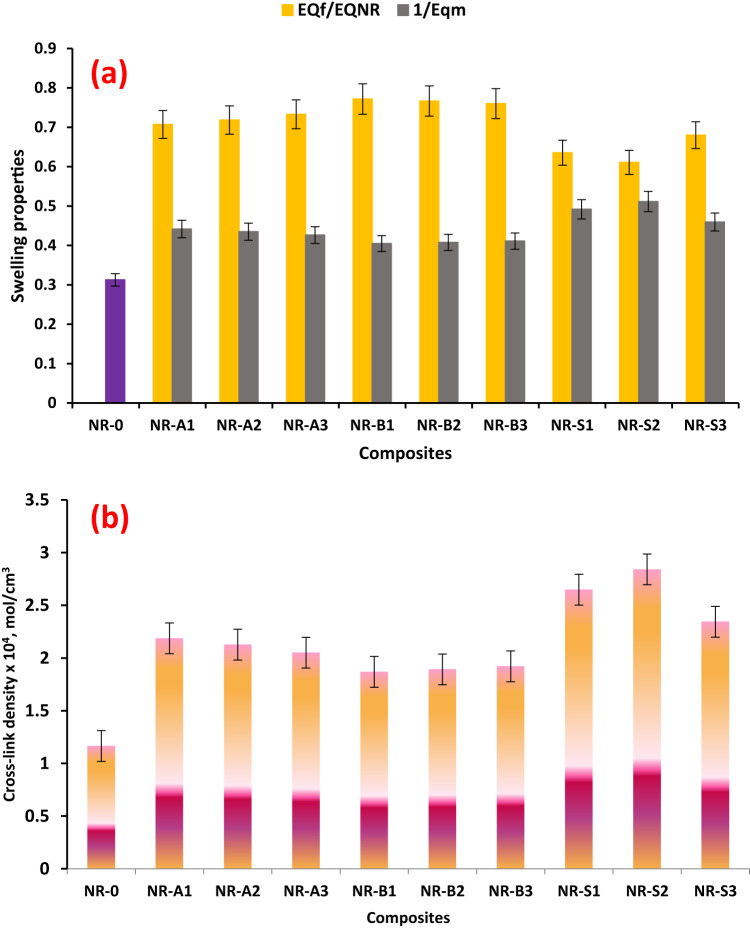
(**a**) Swelling properties of NR composites, (**b**) Crosslinking density of NR composites.

Cross-links between the rubber chains of unfilled natural rubber inhibit swelling by preventing the chains from stretching and diffusing. Through their interactions with the natural rubber, the fillers serve as additional cross-links. Therefore, the measurement results will be considered qualitative only, and it is risky to extrapolate the swelling ratio to the matrix network chain density^48^. Higher cross-link density in vulcanized rubber leads to better thermal stability because vulcanite thermal breakdown requires higher activation energy values^49^. The impact of the interaction between NR and fillers can be computed using the value (1/EQ_m_)^50^. The higher the (EQ_**F**_/EQ_NR_) values, the less contact there occurred between the filler and the rubber matrix. Using 10 phr of Al(LSF) hybrid filler, 15 phr of silica, and 20 phr of Na-bentonite produced the lowest value of (EQ_**F**_/EQ_NR_) and the highest value of (1/EQ_m_), as shown by the results in Fig. 8(a). This suggests a higher NR-filler interaction at these concentrations. Natural rubber, on the other hand, is non-polar, has little oil resistance, and dissolves readily in nonpolar solvents like toluene. Through immersion in toluene, the swelling characteristics of the examined fillers/NR composites were ascertained. The fillers/NR composites under investigation demonstrate how the type of filler affects the swelling coefficient. Toluene’s penetration into the filler-filled NR rubber composites was found to be decreased by the addition of 10 phr of Al(LSF). The more effectively the Al(LSF) interacts with the NR rubber matrix^1^. The EQ_**F**_/EQ_NR_ swelling ratio decreases. Additionally, extra researchers have corroborated this finding in^2^. The Flory-Rehner relation equation’s calculation of the molecular weight between cross-links (Mol.C) based on equilibrium swelling measurements explained these results, which are the outcome of the production of cross-links.

Similarly, the extra physical and chemical cross-links at these concentrations raise the value of cross-link density (νden) (Fig. 8(b)). Moreover, the reason for the rise in the presence of 10 phr of Al(LSF) is that it serves as a bridge to connect the prepared filler to the NR matrix and as reinforcing filler^51^. On the other hand, stronger adherence between these filler particles and the NR matrix is encouraged by the presence of 10 phr of Al(LSF), 15 phr of silica, and 20 phr of Na-bentonite. Better use of the reinforcing action of the studied fillers at this concentration and more efficient stress transfer are made possible by this improved adherence. Because the researched filler is more successfully incorporated into the NR rubber matrix, it improves reinforcing and structural integrity, resulting in a higher crosslinking density. The solubility of the solvent molecule and diffusivity both play a role in the swelling process of the natural rubber matrix, which is a rate-dependent process. In the investigated filler-reinforced natural rubber compound, reduction equilibrium swelling values are attained when the filler is placed into the rubber matrix, resulting in a smaller amount of solvent being absorbed. The explanations for this decrease in swelling near the filler surface are more networking forms between the filler surface and the natural rubber matrix^52,53^.

#### Variation of conformational entropy (ΔS) and elastic Gibbs free energy (ΔG) of NR composites

The investigated thermodynamic effect was applied to identify the interaction between natural rubber and the Al(LSF) hybrid filler, silica, and Na-bentonite in the composite. The computed values of thermodynamic parameters, such as ΔG and ΔS, are shown in Table 3. The Flory-Huggins equation yields an estimate of the elastic Gibbs free energy (ΔG), which is:

**Table 3:** The influence of investigated fillers/ NR composites on the swelling properties.

Ingredient (phr)	NR_0_	NR-A_1_	NR-A_2_	NR-A_3_	NR-B_1_	NR-B_2_	NR-B_3_	NR-S_1_	NR-S_2_	NR-_3_
ΔG, (J/mol))	− 28.6	− 55.9	− 53.1	− 52.2	− 47.3	− 47.9	− 48.7	− 68.3	− 73.5	− 60
ΔS (J/mol.K)	0.096	0.188	0.178	0.175	0.159	0.161	0.164	0.229	0.247	0.156

8 $$\Delta {\text{G }} = {\text{ RT }}\left\{ {{\text{ln }}\left( {{\text{1}} - \phi {\text{r}}} \right){\text{ }} + {\text{ }}\phi {\text{r }} + {\text{ }}\mu {\text{ }}\phi {\text{r}}^{{\mathbf{2}}} } \right\}$$

The natural rubber-solvent interaction parameter (0.393) is represented by µ, and the absolute temperature (298 k) is denoted by T. The universal gas constant (8.314 J.mol^− 1^.K^− 1^) is represented by R, where ϕr is the volume fraction of the natural rubber matrix during the swelling phase, which may be calculated using equilibrium swelling data. Based on rubber statistical theory, the elastic Gibbs free energy (ΔG) and conformational entropy (ΔS) of various natural rubber composites are related by the following equation: - ΔG/T = ΔS. At this point, it is anticipated that the interior energy of the rubber network will not change while extending^54,55^. The conformational Entropy (ΔS) of the NR/10 phr Al(LSF), or 15 phr of silica, and NR/20 phr Na-bentonite is larger than that of natural rubber with filler or other filler loading quantities that have been studied (Table 3). The main reason for the higher ΔS values in NR composites containing the mentioned concentrations is their homogeneous dispersion throughout the rubber matrix.

Again, the elastic properties of natural rubber composites are strongly correlated with their ΔG values^56^. NR/10 phr Al(LSF), NR/20 phr Na-bentonite, and NR/15 phr silica all display higher elastic behavior than other content of investigated fillers, as demonstrated by the value of ΔG. The better elastic performance of the NR receiving Al(LSF) complex is due to the increased compatibility between the NR matrix and the examined filler. Similarly, ΔG values become more negative as the content of the Al(LSF) decreases (10 phr Al(LSF)complex) because the hybrid filler causes the dispersed phase size to shrink and increase the interfacial area up to a certain point^21,57^.

#### Effect of periodic stress-strain on physical properties

As noted by Mathew et al.^58^ and Helaly et al.^47^, the elastic modulus was influenced by several variables that depended on the surface reactivity, including particle size and shape, filler structure, and dispersion in natural rubber chains. The following is an illustration of the relationship between hysteresis and strength using the simple empirical law.

9 $$\:E_{b} = B\left( {Ed_{b} \sum \: _{b} } \right)^{{{\raise0.5ex\hbox{$\scriptstyle 1$} \kern-0.1em/\kern-0.15em \lower0.25ex\hbox{$\scriptstyle 2$}}}}$$.

**Fig. 9:**
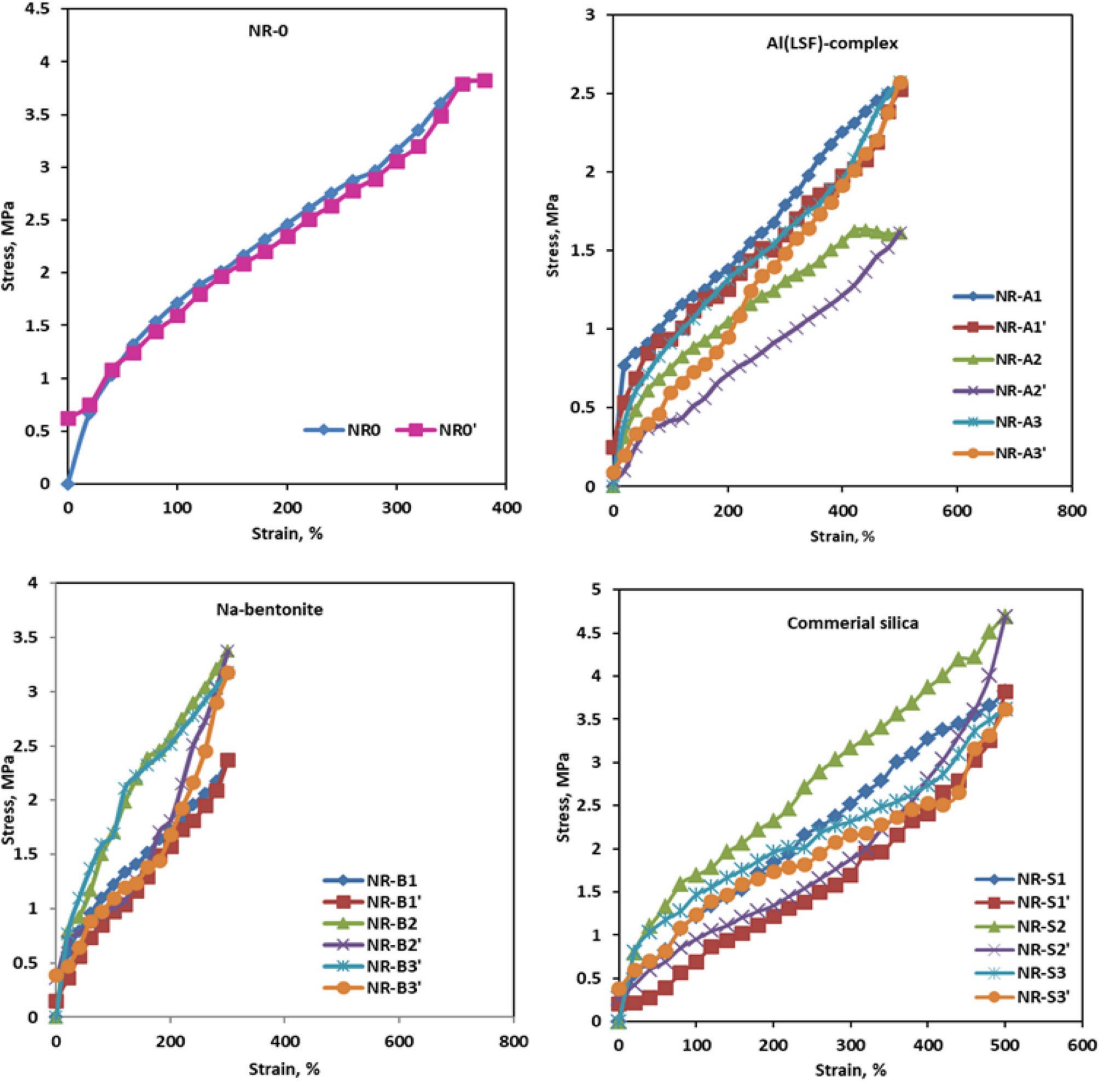
Hysteresis loop for natural rubber loaded with various contents of investigated filler.

Where $\:{Ed}_{b}$ is the energy expended in the deformation before the break (area of the hysteresis loop), $\:{\sum\:}_{b}$is the strain at break, and B is a constant.$\:\:{E}_{b}\:$is the energy input per unit volume to break the tested sample. For each investigated NR composite, stress-relieving or periodic distortions were carried out up to 500% for Al(LSF) hybrid filler/NR and silica/NR, while 300% of the elongation was for Na-bentonite/NR. Figure 9 displays the hysteresis curves for each sample as well as the cyclic stress-strain curves at room temperature (25 + 273 K). Figure 9 also displays the hysteresis loop zone values for each NR vulcanizate’s first stress-strain cycle. These results demonstrate that the NR-A1 (10 phr) sample had a smaller hysteresis loop area of Hls (35.28 MJ) in comparison to other filler-examined samples. The utilization of the Al(LSF) hybrid filler, which can enhance filler-matrix interactions, could be the cause of this. The greater hysteresis loop area, as determined by cyclic distortion, reflects the degree of energy deficit and, thus, the accumulation of heat^21^. The smallest area, where Hls is reduced, indicates the stability of the Al(LSF) (10 phr)/NR composites. That is, the hysteresis is dependent on the type of fillers under study, as illustrated in Fig. 9.

### AC electrical conductivity studies

**Fig. 10:**
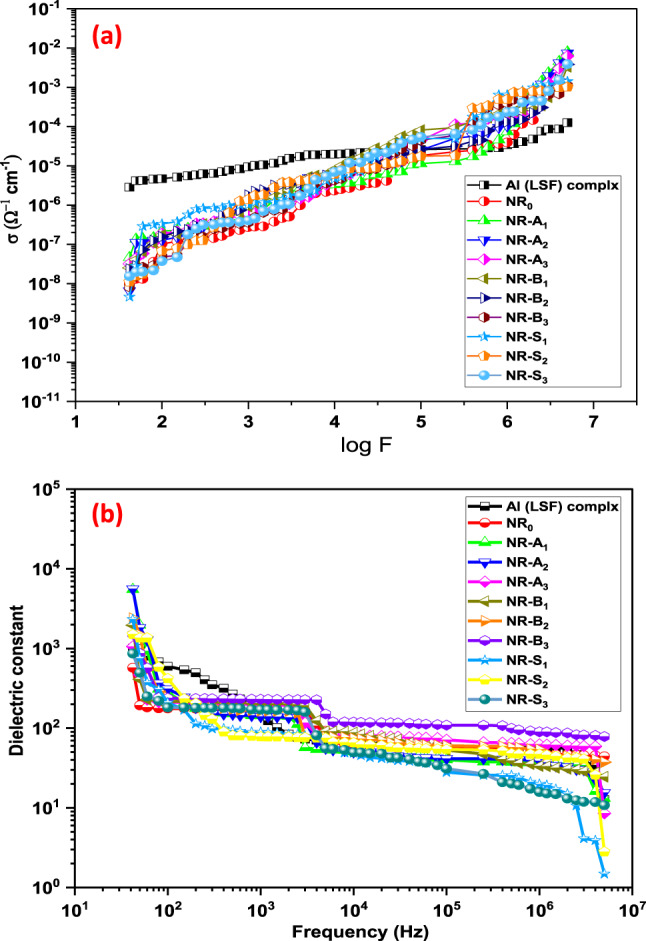
(**a**) Variations of the electrical conductivity with log frequency (f) of NR. (**b**) Represents the relationship between the dielectric constant and frequency for NR composite pellets at room temperature.

The variation of AC conductivity as a function of frequency at room temperature for the pellets labeled Al(LSF), NR_0_, NR-A_1_, NR-A_2_, NR-A_3_, NR-B_1_, NR-B_*2*_, NR-B3, NR-S_1_, NR-S_2_, and NR-S_**3**_ is shown in Fig. 10(a). The electrical conductivity of all samples increased with an increase in applied frequency, which can be related to either the charge-hopping mechanism or a drop in the dielectric constant^59^. The AC conductivity of all the samples was observed to increase as compared to the host matrix polymer. However, NR matrix doping with Al^3+^ ions Al(LSF) complex possesses a high potential, or a positive effect, on electrical conductivity at all frequencies as compared to NR_0_ (control). However, at higher Al concentrations, the electrical conductivity drops down even if charge carrier concentration is increased in the individual NR. According to normal doping rules, the substitution of Al^3+^ at the NR sites creates one extra free carrier in the process and occupies NR sites up to the solubility^60^. Therefore, an increase of carrier concentration up to the solubility limit is logical, but beyond the solubility limit, a new phase of Al_2_O_3_ arises, and therefore substitution of Al is no longer as effective as before^61^.

In contrast, the electrical conductivity values of NR composites at all frequencies are decreased with the doping of Na-bentonite and silica compared to the control. The conductivity at room temperature appears to be promoted by doping Al^3+^in the Al(LSF) complex, which also appears to have improved the defect chemistry and concentration of charge carriers in pure NR.

### Dielectric measurements

As presented in Fig. 10(b) at a frequency of 42 Hz, the dielectric constant of the NR0 sample (control) was found to be 572. The dielectric constant value of the NR composites filled with Al(LSF) complex is dramatically raised by 862% and 874%, respectively, from 572 to 5503 and 5599 (at the same frequency). The inclusion of Al ions into the NR lattice resulted in further increases in the dielectric constant, which reached 5599 (42 Hz). Conversely, the dielectric constant only increases to 2395 upon the implantation of silica and Na-bentonite, showing little effect on the value. The dielectric constant also exhibits a noteworthy improvement in the case of (Al^3+^) doped NR, rising to 5599^14,57^. The value of the dielectric constant is arranged in the following order: NR-A_1_ > NR-A_2_ > NR-B_**2**_ > NR-S_1_ > NR-B1 > NR-S_2_ > NR-B_3_ > NR-A_**3**_ > NR-S_3_ > NR_**0**_ (as shown in Fig. 10(b). The findings verify that doping is positively affecting NR’s dielectric constant. According to the Maxwell-Wagner model, which describes the performance observed for many metal oxides, the dielectric constant dropped as the applied frequency increased^62^. The high periodic reversal of the field at the interfaces is probably the cause of the reductions in permittivity of the produced pellets with increasing applied frequency. The charge carriers’ contribution to the dielectric constant decreased as a result.

## Conclusions

In this study, flexible electric composites evolved by incorporating Al(LSF) hybrid filler into natural rubber. Al(LSF) hybrid filler was effectively obtained from rice straw pulping black liquor, and its structure was confirmed via FTIR and elemental analysis. SEM/TEM pictures revealed that the Al(LSF) had particle sizes between 25 and 40 nm and that the dark platelet-shaped lignin particles interacted with brilliant spherical silica particles. XRD studies revealed the formation of a novel crystalline matrix that results from crosslinking interactions between silica, lignin, and aluminum. NR composites containing the Al(LSF) hybrid filler were prepared, and their properties were compared to those of silica/NR and Na-bentonite/NR composites at varying filler concentrations. The SEM images of the NR composite surfaces suggested that the Al(LSF) hybrid filler particles were well distributed throughout the matrix. The thermal stability of the composites was found to increase with filler loading, mirroring the behavior seen in the Al(LSF)/NR composites. The curing behavior of Al(LSF)/NR composites demonstrated significantly reduced scorch and optimum cure times and increased minimum and maximum torque. The addition of 10 phr of Al(LSF) hybrid filler nanoparticles resulted in significant enhancements in mechanical properties, with tensile strength increasing by about 49% (from 10.3 MPa to 15.39 MPa) and elongation at break soaring by 145% (from 811 to 1987%). The composites containing nanoparticle Al(LSF) exhibit greater DC conductivity than the other micro-counterparts. This bio-based filler provides a sustainable and cost-effective alternative filler to NR composites by utilizing low-cost, commonly available agricultural waste to develop flexible conductive composites.

## Data Availability

All Data is provided within the manuscript.
